# ^18^F-FDG PET/MR versus MR Alone in Whole-Body Primary Staging and Restaging of Patients with Rectal Cancer: What Is the Benefit of PET?

**DOI:** 10.3390/jcm9103163

**Published:** 2020-09-29

**Authors:** Yan Li, Laura Isabel Mueller, Jan Peter Neuhaus, Stefanie Bertram, Benedikt Michael Schaarschmidt, Aydin Demircioglu, Johannes Maximilian Ludwig, Julian Kirchner, Christoph Rischpler, Ken Herrmann, Onofrio Antonio Catalano, Lale Umutlu

**Affiliations:** 1 Department of Diagnostic and Interventional Radiology and Neuroradiology, University Hospital Essen, University of Duisburg-Essen, 45147 Essen, Germany; laura.mueller@uk-essen.de (L.I.M.); benedikt.schaarschmidt@uk-essen.de (B.M.S.); aydin.demircioglu@uk-essen.de (A.D.); johannes-maximilian.ludwig@uk-essen.de (J.M.L.); Lale.Umutlu@uk-essen.de (L.U.); 2 Department of General, Visceral and Transplantation Surgery, University Hospital Essen, University of Duisburg-Essen, Hufelandstrasse 55, 45147 Essen, Germany; jan.neuhaus@uk-essen.de; 3 Institute of Pathology and Neuropathology, University Hospital Essen, University of Duisburg-Essen, Hufelandstrasse 55, 45147 Essen, Germany; stefanie.bertram@uk-essen.de; 4 Department of Diagnostic and Interventional Radiology, Medical Faculty, University Dusseldorf, 40225 Dusseldorf, Germany; julian.kirchner@med.uni-duesseldorf.de; 5 Department of Nuclear Medicine, University Hospital Essen, University of Duisburg-Essen, Hufelandstrasse 55, 45147 Essen, Germany; christoph.rischpler@uk-essen.de (C.R.); ken.herrmann@uk-essen.de (K.H.); 6 Department of Radiology, Massachusetts General Hospital, Harvard Medical School, 55 Fruit Street, White 270, Boston, MA 02114, USA; onofriocatalano@yahoo.it

**Keywords:** rectal cancer, diagnosis, positron-emission tomography, PET, PET/MR, hybrid imaging

## Abstract

Background: To investigate and compare the diagnostic performance of ^18^F-Fluorodeoxyglucose (^18^F-FDG) PET/MR and MR alone in whole-body primary staging and restaging of patients with rectal cancer. Methods: A retrospective analysis was performed to evaluate diagnostic accuracies of combined reading of PET/MR and MR alone in T, N and M staging against the reference standard. Inter-observer agreement regarding TNM staging was calculated separately for PET/MR and MR alone. Results: A total of 39 studies of 34 patients could be evaluated. Diagnostic accuracies of PET/MR and MR alone were the same in locoregional T staging. For predicting N+ stage, the specificity of combined reading of PET and MR (0.917 and 0.833 for reader 1 and 2, respectively) was slightly higher than MR alone (0.833 and 0.75) without significantly increasing the overall accuracy (0.783 vs. 0.783 and 0.783 vs. 0.739). For detecting distant metastasis, the sensitivities of PET/MR and MR alone were shown equal (1.0 vs. 1.0 and 0.938 vs. 0.938), while the specificity of PET/MR was marginally lower (0.87 vs. 0.913 and 0.826 vs. 0.87). The inter-observer agreements were good to excellent in M (κ = 0.64 and 0.637 for PET/MR and MR alone, *p* < 0.001) and N staging (0.819 and 0.738, *p* < 0.001).Conclusion: PET did not yield a significant improvement in diagnostic accuracy of PET/MR in TNM staging of rectal cancer, since MR alone facilitated accurate classification of disease stage with good to excellent inter-observer agreement.

## 1. Introduction

With an estimated 500,000 new cases and 243,000 deaths in 2018, colorectal cancer was the second most common cancer and the second leading cause of cancer-related death in Europe [1]. Precise assessment of locoregional and distant tumor extent is of paramount importance for treatment planning and prognosis prediction. Rectal MR comprising a dedicated imaging protocol is mandatory and is the first-choice technique to evaluate locoregional tumor stage in both primary staging and restaging after chemoradiotherapy (CRT) [2]. Chest and abdomen CT are recommended as part of the staging protocol for metastasis detection. Liver MR with hepatocyte-specific contrast agent is indicated in indeterminate liver lesions found in CT [3,4]. Furthermore, cost-effective contrast-enhanced ultrasound that can improve the characterization of indeterminate solid hepatic lesions presented on CT should also be considered [5,6]. Therefore, it can be quite burdensome for patients to undergo several imaging modalities at different time points and experience exposure to different contrast agents.

Whole-body ^18^F-fluorodeoxyglucose (^18^F-FDG) PET/MR with dedicated rectal and liver MR protocols represents a promising imaging tool for both locoregional and whole-body staging of rectal cancer in a single session, resulting in a markedly improved diagnostic workup [7]. Moreover, whole-body PET/MR has been shown to provide added value to CT imaging in metastasis detection and characterization of equivocal lesions, which might potentially obviate additional imaging workup [8,9]. PET/MR has also yielded superior diagnostic accuracy to PET/CT at the time of diagnosis and during follow-up, and might alter the clinical management prompted by standard imaging modalities [10,11,12]. Nevertheless, while hybrid imaging has been shown to be superior to conventional CT imaging, the added value of using PET in MR imaging remains ambiguous.

Hence, the primary aim of our study was to investigate and compare the diagnostic performance of ^18^F-FDG PET/MR and MR alone in TNM staging of patients with rectal cancer to assess the added value of PET to MR diagnostics. Secondly, we evaluated additional findings detected on whole-body PET/MR and their impact on patient management.

## 2. Materials and Methods

### 2.1. Patient Selection

Our study was a retrospective single center analysis approved by the institutional review board. Informed consent was waived due to retrospective design. Inclusion criteria comprised (a) histopathologically confirmed diagnosis of a rectal malignant tumor within 16 cm from the anal verge, (b) ^18^F-FDG PET/MR as an imaging modality for primary staging or restaging and (c) available histopathologic tissue sampling of lesions suspicious for metastases found in PET/MR or imaging follow-up (CT, MR or PET/MR) after three months. Exclusion criteria were inadequate imaging quality and absence of imaging follow-up.

The indications for PET/MR were categorized as (1) primary staging in untreated patients; (2) preoperative restaging after neoadjuvant CRT; (3) restaging by clinical or labor indices of tumor recurrence/distant metastasis in patients under post-treatment surveillance.

### 2.2. ^18^F-FDG PET/MR Imaging Protocol

All patients underwent PET/MR examinations on a whole-body 3.0 tesla PET/MR scanner (Biograph mMR, Siemens Healthcare, Erlangen, Germany). After a fast period of at least 6h (average blood glucose level = 102 mg/dL), body-weight adapted ^18^F-FDG (average value ± standard deviation = 266.6 ± 58.8 MBq) was administered intravenously 1h prior to the scan. For primary staging and preoperative restaging, the rectum was cleansed by patients themselves with clyster (Microlax^®^, Johnson & Johnson, Neuss, Germany) and after that was filled with 50 mL ultrasound-gel directly before the scan began. The whole-body PET/MR protocol covered from the skull base to the proximal femur and comprised a dedicated rectal MR protocol with high-resolution T2-weighted (T2w) imaging and diffusion-weighted imaging (DWI) according to the latest recommendations of the European Society of Gastrointestinal and Abdominal Radiology [2,13]. For M staging liver MR protocol, dynamic contrast-enhanced T1-weighted (T1w) imaging with non-hepatobiliary contrast agent (gadoterate meglumine, Dotarem^®^, Guerbet, France) and the dedicated whole-body protocol were applied. Detailed PET/MR imaging protocol and MR sequences are summarized in Supplementary Materials (Table S1).

### 2.3. Imaging Analysis and Interpretation

Two board-certificated radiologists (YL and BS, both with 5 years of experience in oncological hybrid imaging) analyzed PET/MR images independently utilizing a dedicated post-processing software (Syngo.via, VB30B, Siemens Healthcare, Erlangen, Germany). Readers were only aware of the clinical indications.

In untreated and preoperative post-CRT patients, each reader evaluated the locoregional tumor stage using T2w imaging and DWI at first. The results were classified in T and N stages (TNM 8th edition) and simplified as T0-2/T3-4 and N0/N+. The T stage was determined only on T2w imaging [2]. The hyperintense presence of the tumor was also assessed in the *b*1000 of DWI. The chosen MR criteria for malignant lymph node (N+) were: size ≥ 5 mm, irregular or indistinct borders, and heterogeneous signal intensity on T2w imaging [14,15]. In a second step, the readers rated M stage as M0 or M1 with only MR images, including dedicated liver MR and the whole-body protocol. Lastly, the readers re-evaluated the TNM stages using additional PET information and fused images of PET and MR. In patients after rectal resection and under surveillance, the readers reviewed merely M stage concerning distant metastasis and/or local recurrence in a similar fashion, first using MR and then combined reading of PET/MR images.

In a consensus meeting, two readers and one nuclear physician measured maximum standard uptake value (SUVmax) of FDG uptake within the rectal tumor and calculated SUVmax_Ratio (SUVmax of tumor relative to liver). Detailed methods of measurement are summarized in the Supplementary Materials. Additional findings on PET/MR were documented and further classified.

### 2.4. Reference Standard

For T and N staging, histopathologic findings of surgical specimens were used as the reference standard. In untreated patients, who underwent PET/MR before neoadjuvant CRT, the information on T and N stages at the time of PET/MR could be derived retrospectively from the histopathologic results of resection specimens that suggested initial tumor extent. For M stage, histopathologic findings of metastasis-suspect lesions in PET/MR were the reference standard. In the case of unavailable histopathology, imaging follow-ups served as reference. A size increase or decrease in accordance with change of other metastasis-suspect lesions indicated the presence of metastasis. If no metastasis presented in initial PET/MR and imaging follow-ups, the M stage was determined as M0 at the time of PET/MR.

### 2.5. Statistics

Descriptive analysis with contingency tables was used to calculate the sensitivity, specificity and overall accuracies of combined reading of PET/MR and MR alone in TNM staging against the reference standard. Cohen’s kappa was calculated to determine interobserver-agreement between readers. Median values of SUVmax and SUVmax_Ratio of tumor in post-CRT patients with ypT1-2 and ypT3-4 stadiums were compared with each other using Mann–Whitney *U* test. A *p*-value < 0.05 was considered statistically significant. All statistical tests were performed using SPSS (Statistical Package for the Social Sciences, version 25, IBM, Armonk, NY, USA).

## 3. Results

### 3.1. Patient’s Characteristics

From 2012 to 2020, thirty-four patients meeting the inclusion criteria were retrospectively enrolled. The mean age was 58 years (range 28–78) and 17 patients were females (50%). Five patients underwent PET/MR twice, resulting in a total of 39 studies. A total of 9 studies were performed for primary staging; 14 studies for preoperative restaging after CRT; and 16 studies for restaging due to indices of local recurrence or metastasis in patients under post-treatment surveillance. Three patients underwent PET/MR at two time points for primary staging and preoperative restaging after CRT; another 2 patients underwent PET/MR for primary staging and restaging after rectum resection and with clinical indication of tumor recurrence. Patient characteristics are summarized in Table 1.

### 3.2. Diagnostic Performance of PET/MR and MR Alone in Locoregional T and N Staging

Out of the 39 studies, 23 studies of patients with primary rectal cancer were included for T and N staging analysis, comprising 9 for primary and 14 for preoperative post-CRT restaging. According to histopathology, 16 studies showed T3-4 stages (70%). Using T2w imaging, the overall sensitivity, specificity, and accuracy in predicting T3-4 stages were 0.75, 0.857, 0.783 for reader 1 and 0.625, 0.429, 0.565 for reader 2, respectively (Table 2). Out of the 9 untreated patients, 2 presented with T2 stage, 6 with T3 stage and 1 with T4 stage. The diagnostic results of both readers in predicting T3-4 stages were identical (κ = 1, *p* < 0.001) with a sensitivity of 0.857, a specificity of 0.50, and accuracy of 0.778. All rectal tumors appeared hyperintense in DWI.

In the 14 post-CRT patients, 9 patients showed ypT3, 4 ypT2 and 1 ypT0. In predicting ypT3-4 stage, the diagnostic performance (sensitivity 0.667, specificity 1.0, accuracy 0.786 for reader 1; 0.44, 0.40, 0.429 for reader 2) and inter-observer agreement (κ = 0.29, *p* > 0.05) were markedly reduced. Hyperintense appearance of the tumor in DWI was present in 7 out of 9 ypT3 and in only 1 out of 5 ypT0-2 stages. Combining the additional PET information with T2w imaging, the determination of T stages in each patient remained the same for both readers (overall accuracies in predicting T3-4 stages 0.75 for reader 1 and 0.625 for reader 2).

A total of 11 out of the 23 studies presented N+ stages (48%). Using T2w images, reader 1 correctly diagnosed 8 N+ cases with a sensitivity of 0.727 (8/11), specificity of 0.833 (10/12), and accuracy of 0.783 (18/23); with a combined reading of PET and T2w images, the specificity could be increased to 0.917 (11/12) and the sensitivity was reduced to 0.636 (7/11) with the same accuracy of 0.783 (18/23) (Table 3). For reader 2, sensitivity was the same of 0.727 (8/11), specificity of 0.75 (9/12) and accuracy 0.739 (17/23); with additional information from PET, the specificity was increased to 0.833 (10/12) and the sensitivity was unchanged with a marginally enhanced accuracy of 0.783 (18/23).

### 3.3. Diagnostic Performance of PET/MR and MR Alone in M Staging

According to the reference standard, M1 stages (distant metastasis and/or local recurrence) were present in 16 of the 39 studies (41%). A total of 12 out of the 16 cases were histologically proven. The metastatic locations comprised 9 metastases in the liver, 4 in the lung, 2 in presacral local recurrence and 1 in a distant lymph node. Reader 1 correctly diagnosed all cases with MR alone (sensitivity 1.0, specificity 0.913 and accuracy 0.949; Table 4). With combined reading of PET, sensitivity remained the same and specificity was slightly reduced (1.0, 0.87, 0.923). The results of reader 2 were similar (0.938, 0.87, 0.897 using MR; 0.938, 0.826, 0.872 using PET/MR) and inter-observer agreement demonstrated a good concordance (κ = 0.69 for MR and 0.692 for PET/MR, both *p* < 0.001).

### 3.4. Metabolic Information of Untreated and Post-CRT Rectal Tumor

The median time interval between post-CRT PET/MR and surgical resection was 10 days (range 5–94). The mean values of SUVmax and SUVmax_Ratio of tumor in post-CRT ypT3-4 stages were higher than in ypT0-2 but without a significant difference (Table 5). Within 9 patients with ypT3-4 stages, 6 showed SUVmax_Ratio > 1; within 5 patients with ypT0-2 stages, 2 showed SUVmax_Ratio > 1.

### 3.5. Additional Findings and Their Clinical Management

**Figure 1:**
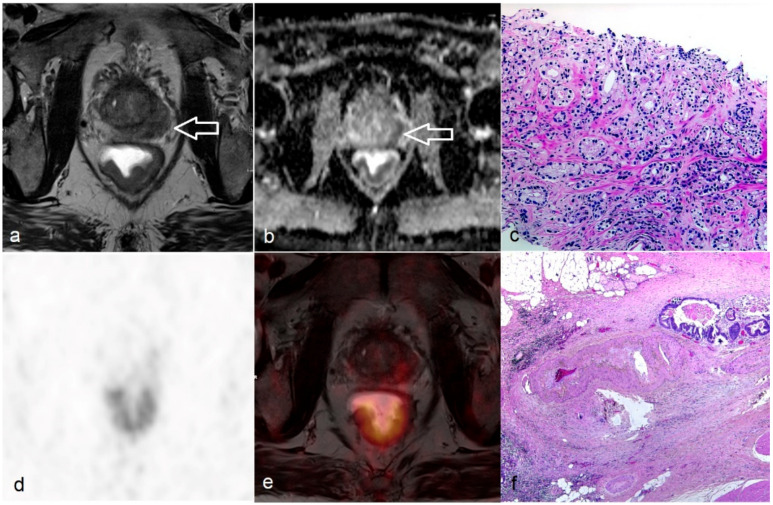
Additional finding of prostate cancer in a 71-year-old male with rectal cancer. (**a**) T2-weighted high-resolution imaging showing rectal cancer at the posterior circumference and concomitant presence of a tumor-suspect lesion at the left peripheral zone of prostate (white arrow); (**b**) Apparent diffusion co-efficient map reveals restricted diffusion in both tumors (white arrow heading prostatic tumor) and indicates the presence of malignancies; (**c**) Prostate biopsy shows prostatic adenocarcinoma with closely packed small-sized glands; (**d**) Pelvic PET demonstrates intense ^18^F-fluorodeoxyglucose metabolism of the rectal cancer and low uptake in the prostate cancer; (**e**) Fused imaging of T2-weighted image and PET; (**f**) Surgical specimen shows rectal adenocarcinoma infiltrating through the tunica muscularis propria into perirectal fat tissue.

**Figure 2:**
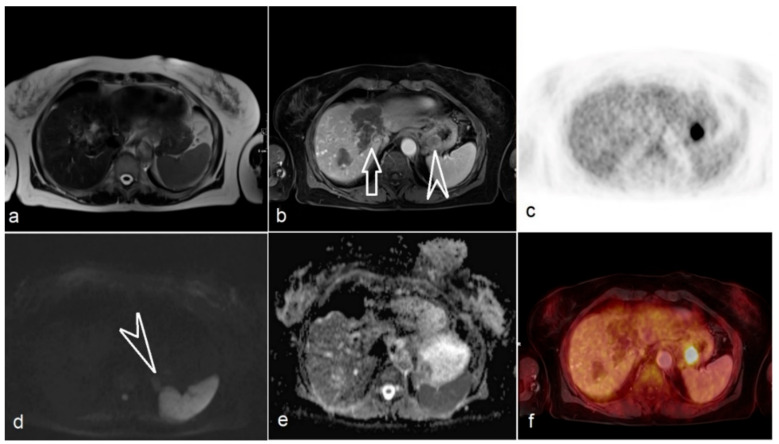
Additional finding of gastrointestinal stromal tumor in a 61-year-old female with hepatic metastasis of rectal cancer. (**a**) T2-weighted imaging of the liver; (**b**) Contrast-media-supported T1-weighted imaging shows necrosis of liver metastasis (white arrow) and incidental tumor mass in the stomach (arrow head); (**c**) PET shows intense ^18^F-fluorodeoxyglucose uptake of the histopathologically diagnosed gastrointestinal stroma tumor and missing uptake in the liver metastasis; (**d**) *b*1000 of diffusion-weighted imaging demonstrates the slightly hyperintense appearance of the stomach tumor (arrow head); (**e**) Apparent diffusion co-efficient map; (**f**) Fused image of T1-weighted image and PET.

Additional findings were present on PET/MR in 3 patients (9%). In a 71-year-old male for primary staging of rectal cancer with initial T3N0 stage, a tumor-suspect lesion in the left peripheral zone of the prostate could be detected in T2w and DWI (Figure 1). This lesion was biopsied and proven as prostate adenocarcinoma, which was treated with high-dose-rate brachytherapy with 13.1Gy one week after accomplishing neoadjuvant CRT (radiation dose of 50.4Gy combined with fluorouracil) of the rectum. A 61-year-old female with hepatic metastasis after rectal resection (pT3N0) and adjuvant chemotherapy (FOLFOX regimen plus Bevacizumab) underwent PET/MR for follow-up assessment and planning of metastasis resection. Based on PET/MR a suspicious lesion in the stomach fundus with high metabolic activity could be detected (Figure 2), which was missed by both readers in reviewing MR-only images. This lesion was resected at the time of extended right hemihepatectomy and was histopathologically diagnosed as a gastrointestinal stromal tumor. In accordance to PET, histopathology revealed complete necrosis of liver metastasis and absence of vital tumor cells. In another 50-year-old female for primary staging with initial T2N0M0 stage, a meningioma typical lesion of 16 mm without pathologic FDG-uptake around the cerebellum could be depicted on MR images. Due to the absence of clinical symptoms, a watchful waiting concept was chosen. No change in lesion diameter was present in the latest MR follow-up after 3 years.

## 4. Discussion

In this retrospective analysis, we compared the diagnostic performance of ^18^F-FDG PET/MR with MR alone in TNM staging of patients with rectal cancer. With added information from PET, the diagnostic accuracy of PET/MR was not significantly improved in locoregional (T and N stages) or distant tumor assessment (M stage), since MR alone yielded accurate classification of disease stage with good to excellent inter-observer agreement. As demonstrated in our study, PET may play a role in depicting additional extrahepatic malignancies and may potentially affect the treatment plan. Our results also revealed the feasibility of whole-body ^18^F-FDG PET/MR as “one-stop shop” imaging tool for precise assessment of local and distant disease extent, which obviates additional diagnostic workup and improves patient satisfaction.

A dedicated rectal MR protocol containing high-resolution T2w images and DWI was pivotal for accurate assessment of tumor extent and treatment-planning [16]. The diagnostic accuracy for predicting T3-4 stages of untreated patients using T2w imaging amounted to 0.778 with excellent inter-observer agreement (κ = 1). Both readers overstaged the local tumor extent in one of the two patients with T2 stage at primary staging due to misinterpretation of the desmoplastic reaction at the tumor border as extramural tumor invasion, which is a common and well-known inevitable staging failure [17,18]. The sensitivity of T2w imaging in patients after CRT was markedly reduced from 0.857 to 0.667 and 0.44 for both readers. This result was in line with a meta-analysis in 1556 post-CRT patients with rectal cancer, which estimated a pooled sensitivity of only 0.553 (95% CI: 0.40–0.697) in differentiating between T0-2 and T3-4 stages [19]. We agree with the authors that restaging in post-CRT patients is challenging because of the inherent limitation of T2w imaging in distinguishing post-treatment fibrotic areas from residual tumor at the interface of the tumor border to mesorectal fat. Due to the lower spatial resolution of PET and overlap of tumor glucose metabolism between post-CRT yT0-2 and yT3-4 stages in our study, the combined reading of PET and T2w did not help improve the diagnostic accuracy. Taking liver metabolism as background activity, the SUVmax_Ratio of tumor after CRT could not reliably differentiate yT3-4 from yT0-2 stages (sensitivity 0.67, specificity 0.6 and accuracy 0.64). This limitation of PET and T2w may be overcome by the benefit of DWI, which could better predict histopathologic viable tumors from fibrotic scarring [20]. Accordingly, our results also suggested the superior sensitivity of 0.778 for DWI to depict non-downstaged patients with yT3-4 stage and higher specificity of 0.8. Incorporating both size and morphological criteria determined by the expert panel [2,14], T2w imaging alone resulted in a moderate overall accuracy (0.783 and 0.739 for reader 1 and 2, respectively) in predicting N+ stage. Using the same diagnostic criteria, the sensitivity of MR decreased further in post-CRT patients, while the specificity increased to 100%, since residual micrometastasis in a size-reduced lymph node might be beyond the detection limit of conventional MR imaging. Until now, no solid evidence supported the routine clinical use of PET in N staging, because the sensitivity in untreated patients was reported as low as only 0.485 despite the extremely high specificity of 0.939 [21]. In a meta-analysis of PET and PET/CT in 409 colorectal cancer patients, the estimated results confirmed again the limited sensitivity of 0.429 (95% CI: 0.36–0.5) and high specificity of 0.879 (95% CI: 0.826–0.9) for PET [22]. Our results reflected the same findings that combined reading of PET and MR did not improve the sensitivity of MR alone. However, due to the higher specificity of PET, combined reading of PET/MR in our study helped to revise one false-positive result of MR in untreated patients for both readers. The stringent criteria of PET/MR based on size, morphology and ^18^F-FDG uptake increased the detection threshold for lymph node metastasis further. They limited the overstaging rate, which finally helped to avoid unnecessary neoadjuvant CRT and associated potential adverse effects for the patients.

Accumulating evidence indicates the superior diagnostic performance of ^18^F-FDG PET/MR in M staging against standard-of-care imaging (e.g., CT, MR or PET/CT) in colorectal cancer [10,11]. In a recent study that prospectively investigated 71 untreated patients in advanced stage, PET/MR, including dedicated rectal and liver MR, outperformed CT in diagnostic specificity (0.98 vs. 0.73) for M staging, as PET/MR was shown to better assess indeterminate lesions, metastases and incidental findings [9]. Our study results confirmed the high negative predictive value of PET/MR (1.0 and 0.95 for reader 1 and 2) in distant metastasis detection. Nevertheless, false-positive results of PET/MR still occurred in rare cases due to tumor-mimicking inflammatory changes. For instance, both readers misinterpreted one FDG-avid and morphologically diffuse infiltrating lesion as tumor recurrence at the pelvic floor in a patient after CRT and abdominoperineal excision. The surgical specimen was later histopathologically proven as post-radiation tissue necrosis. The nearly identical diagnostic results of MR and PET/MR in predicting M stage in our study might be explained by the small number of patients and lower proportion of patients with M0 stage. Despite that, a potential benefit provided by PET was the predictive value regarding the therapy response of both primary tumor and distant metastasis after CRT [23,24]. In a female patient with gastrointestinal stroma tumor of the stomach as an additional extrahepatic finding (Figure 2), the level of FDG uptake in the central gross liver metastasis was below the average of adjacent liver parenchyma, indicating good response after chemotherapy. Indeed, the histopathology revealed complete necrosis without any residual tumor cells.

There were several limitations in our study. Firstly, it was a retrospective study associated with a selection bias. A high proportion of patients presented with advanced disease stage with locoregional N+ and/or M+ stages at the time point of PET/MR. Therefore, the previously reported superior diagnostic specificity of combined PET/MR in M staging could not be well reproduced. The heterogeneity of our study cohort, which included both untreated patients and those with neoadjuvant CRT, might also influence the diagnostic results of MR alone and PET/MR. For instance, in 14 post-CRT and preoperative studies, the metabolism of tumor metastasis at the time of PET/MR might be reduced by CRT, which might have led to underperformance of PET in detecting distant and lymphatic metastasis. Secondly, our analysis included only a small sample size of 34 patients and the results may not be generalizable. Thirdly, the initial histopathological T and N stages at the time of primary staging with PET/MR were derived from the final surgical specimens in those patients at advanced stages, for whom preoperative neoadjuvant CRT was necessary. Post-CRT regressive changes could only suggest the initial real locoregional tumor stages. Lastly, because of the limited patient numbers, we did not evaluate local tumor response after CRT, while the potential of PET seemed to be its response evaluation in patients with locally advanced stage [25,26]. Further studies with larger sample sizes are warranted.

## 5. Conclusions

With additional information provided by PET, the diagnostic accuracy of PET/MR was not necessarily improved in TNM staging of rectal cancer, since MR alone facilitated accurate classification of disease stage with good to excellent inter-observer agreement.

## Figures and Tables

**Table 1:** Sociodemographic and clinical characteristics of 34 patients with 39 PET/MR studies.

Characteristics	Value (Percentage)
Sex	
Male	*n* = 17 (50%)
Female	*n* = 17 (50%)
Age	
Mean	58
Range	18–78
Histopathology of rectal cancer	
Adenocarcinoma	*n* = 30 (88.2%)
Mucinous adenocarcinoma	*n* = 4 (11.8%)
Location of rectal cancer	
Upper (12–16 cm from anal verge)	*n* = 7 (20.6%)
Middle (6–12 cm)	*n* = 14 (41.2%)
Lower (≤6 cm)	*n* = 13 (28.2%)
Indications for PET/MR	
Primary staging	*n* = 9 (23.1%)
Preoperative restaging after CRT *	*n* = 14 (35.9%)
Restaging under surveillance with indices of local recurrence/distant metastasis	*n* = 16 (41.0%)

* CRT = chemoradiotherapy.

**Table 2:** Contingency table of diagnostic results of PET/MR and MR in T staging.

		Untreated Patients (*n* = 9)			Post-CRT * Patients (*n* = 14)			All Patients (*n* = 23)		
		T1/2	T3/4	Total Number	T1/2	T3/4	Total Number	T1/2	T3/4	Total Number
Reader 1	T1/2	1	1	2	5	3	8	6	4	10
	T3/4	1	6	7	0	6	6	1	12	13
	Total number	2	7	9	5	9	14	7	16	23
Reader 2	T1/2	1	1	2	2	5	7	3	6	9
	T3/4	1	6	7	3	4	7	4	10	14
	Total number	2	7	9	5	9	14	7	16	23
Inter-observer agreement for T stage		κ **= 1, *p* < 0.001			κ = 0.29, *p* > 0.05			κ = 0.553, *p* = 0.008		

* CRT = chemoradiotherapy; κ ** = Cohen’s kappa. Note: determination of T stages with MR alone (T2w imaging) or with PET/MR (T2w and PET) remained the same for both readers.

**Table 3:** Contingency table of diagnostic results of PET/MR and MR in N staging.

			Untreated Patients (*n* = 9)			Post-CRT * Patients (*n* = 14)			All Patients (*n* = 23)		
			N0	N+	Total Number	N0	N+	Total Number	N0	N+	Total Number
Reader 1	MR alone	N0	4	0	4	6	3	9	10	3	13
		N+	2	3	5	0	5	5	2	8	10
		Total Number	6	3	9	6	8	14	12	11	23
	Combined PET/MR	N0	5	0	5	6	4	10	11	4	15
		N+	1	3	4	0	4	4	1	7	8
		Total Number	6	3	9	6	8	14	12	11	23
Reader 2	MR alone	N0	3	0	3	6	3	9	9	3	12
		N+	3	3	6	0	5	5	3	8	11
		Total Number	6	3	9	6	8	14	12	11	23
	Combined PET/MR	N0	4	0	4	6	3	9	10	3	13
		N+	2	3	5	0	5	5	2	8	1
		Total Number	6	3	9	6	8	14	12	11	23
Inter-observer agreement for N stage			κ ** = 0.768 for MR, *p* = 0.018 κ = 0.780 for PET/MR, *p* = 0.016			κ = 0.689 for MR, *p* = 0.01 κ = 0.837 for PET/MR, *p* = 0.001			κ = 0.738 for MR, *p* < 0.001 κ = 0.819 for PET/MR, *p* < 0.001		

* CRT = chemoradiotherapy; κ ** = Cohen’s kappa.

**Table 4:** Contingency table of PET/MR against reference standard in predicting M stage in 39 studies.

			M Stage (*n* = 39)		
			M0	M1	Total Number
Reader 1	MR alone	M0	21	0	21
		M1	2	16	18
		Total number	23	16	39
	Combined PET/MR	M0	20	0	20
		M1	3	16	19
		Total number	23	16	39
Reader 2	MR alone	M0	20	1	21
		M1	3	15	18
		Total number	23	16	39
	Combined PET/MR	M0	19	1	20
		M1	4	15	19
		Total number	23	16	39
Inter-observer agreement for M stage		κ * = 0.637 for MR and 0.64 for PET/MR, both *p* < 0.001			

κ * = Cohen’s kappa.

**Table 5:** SUVmax and SUVmax_Ratio of rectal tumor at histopathologically different T stages.

	Untreated T0-2 Stage (*n* = 2)	Untreated T3-4 Stage (*n* = 7)	Post-CRT ** T0-2 Stage (*n* = 5)	Post-CRT T3-4 Stage (*n* = 9)
SUVmax	17.25 (± 10.11) (mean ±SD)	15.9 (± 4.5)	2.89 (± 3.76)	7.98 (± 4.88)
			*p* * = 0.125	
SUVmax_Ratio	6.01 (± 4.41)	4.29 (± 1.8)	0.85 (± 0.6)	2.1 (± 4)
			*p* * = 0.142	

* Mann–Whitney *U* test; CRT ** = chemoradiotherapy; SD = standard deviation.

## Supplementary Materials

The following are available online at https://www.mdpi.com/2077-0383/9/10/3163/s1, Table S1: Detailed rectal, liver and whole body MR protocols.
