# Supplementary material for: 18F-FDG PET/MR versus MR Alone in Whole-Body Primary Staging and Restaging of Patients with Rectal Cancer: What Is the Benefit of PET?
Source: J Clin Med. 2020 Sep 29;9(10):3163. doi: 10.3390/jcm9103163 (PMC7599654; doi:10.3390/jcm9103163)
Supplement: Supplementary file 1 [file jcm-09-03163-s001.pdf]

Supplementary Material

Title: ^{18}F-FDG PET/MR versus MR alone in whole-body primary staging and restaging of patients with rectal cancer: what is the benefit of PET?

1. Technical parameters of PET/MR protocols

PET acquisition time per bed-position took 2 minutes and 4 bed-positions were needed to cover from skull basis to proximal femur. PET images were reconstructed using a three-dimensional Ordinary Poisson Ordered Subset Expectation Maximation algorithm (3D OP-OSEM; 3 iterations, 21 subsets, voxel size 2.1 x 2.1 x 2.0 mm3, 3D Gaussian filter of 4.0 mm).

Table 1. Detailed rectal, liver and whole body MR protocols.

**MR sequences**		Plane	Matrix sizes	Slice thickness (mm)	Gap	Field of view (mm)
**Rectal protocol of pelvis**	T2w TSE (a)	sagittal	310 x 320	4	10%	200
	T2w TSE	coronal	310 x 320	3	0	200
	T2w TSE	axial	310 x 320	3	0	160
	EPI DWI (b)	axial	102 x 160	3	0	260
Liver protocol	T1w_3D VIBE (c)	axial	512 x 230	3.5	0	400
	T1w with in and out of phases (d)	axial	256 x 256	7	20%	380
Whole body protocol from skull basis to proximal femur	T2 HASTE (e)	axial	320 x 194	7	20%	400
	T1_3D VIBE (f)	axial	320 x 165	3	0	380
	DWI (g)	axial	160x90	5mm	20%	420

a) a) T2-weighted Turbo spine echo (TSE) without fat suppression.
b) Diffusion weighted echo planar sequence (EPI DWI, B-values: 0, 500 and 1000 s/mm^2).
c) Dynamic contrast-enhanced 3D T1-weighted Volumetric Interpolated Breath Hold Examination (VIBE) with fat-suppression and repetitive scans before and after i.v. injection of 0.2 ml gadoterate meglumine (Dotarem®, Guerbet, France) per kilogram bodyweight (arterial phase with 20sec delay, portalvenous with 50sec delay and venous phase with 80sec delay).
d) T1-weighted gradient echo sequence in phase and out of phase.
e) T2-weighted (T2w) half-Fourier acquisition single-shot turbo spin echo (HASTE)
f) Contrast-enhanced 3D T1-weighted Volumetric Interpolated Breath Hold Examination (VIBE) with fat-suppression.
g) Diffusion weighted echo planar sequence (EPI DWI, B-values: 0, 500 and 1000 s/mm^2).

2. Measurement of SUVmax in tumor and liver

As metabolic parameter, maximum of standard uptake value (SUVmax) of rectal tumor was measured by drawing a spherical volume of interest in the most FDG-avid region of PET. SUVmax of the liver was determined in the same way by placing a larger volume of interest in the right lobe. SUVmax_Ratio was calculated as the ratio of SUVmax of rectal tumor relative to the liver. All measurements were performed in a consensus meeting of two readers and one nuclear physician with dedicated post-processing software (Syngo.via, VB30B, Siemens Healthcare, Erlangen, Germany).
